# Assessment of Emergency Medicine Resident Performance in a Pediatric In Situ Simulation Using Multi-Source Feedback

**DOI:** 10.7759/cureus.16812

**Published:** 2021-08-01

**Authors:** Michael C Nguyen, Nicole C Elliott, Diane P Begany, Katie M Best, Matthew D Cook, Michael R Jong, Zachary M Matuzsan, Louis A Morolla, Suzanne S Partington, Bryan G Kane

**Affiliations:** 1 Department of Emergency and Hospital Medicine, Lehigh Valley Hospital and Health Network/University of South Florida Morsani College of Medicine, Allentown, USA; 2 Department of Pediatrics, Lehigh Valley Hospital and Health Network/University of South Florida Morsani College of Medicine, Allentown, USA

**Keywords:** multi-source feedback, pediatric, emergency medicine, assessment, simulation

## Abstract

Introduction

Multi-source feedback (MSF) is an evaluation method mandated by the Accreditation Council for Graduate Medical Education (ACGME). The Queen’s Simulation Assessment Tool (QSAT) has been validated as being able to distinguish between resident performances in a simulation setting. The QSAT has also been demonstrated to have excellent MSF agreement when used in an adult simulation performed in a simulation lab. Using the QSAT, this study sought to determine the degree of agreement of MSF in a single pediatric (Peds) simulation case conducted in situ in a Peds emergency department (ED).

Methods

This Institutional Review Board-approved study was conducted in a four-year emergency medicine residency. A Peds resuscitation case was developed with specific behavioral anchors on the QSAT, which uses a 1-5 scale in each of five categories: Primary Assessment, Diagnostic Actions, Therapeutic Actions, Communication, and Overall Assessment. Data was gathered from six participants for each simulation. The lead resident self-evaluated and received MSF from a junior peer resident, a fixed Peds ED nurse, a random ED nurse, and two faculty (one fixed, the other from a dyad). The agreement was calculated with intraclass correlation coefficients (ICC).

Results

The simulation was performed on 35 separate days over two academic years. A total of 106 MSF participants were enrolled. Enrollees included three faculty members, 35 team leaders, 34 peers, 33 ED registered nurses (RN), and one Peds RN; 50% of the enrollees were female (n=53). Mean QSAT scores ranged from 20.7 to 23.4. A fair agreement was demonstrated via ICC; there was no statistically significant difference between sources of MSF. Removing self-evaluation led to the highest ICC. ICC for any single or grouped non-faculty source of MSF was poor.

Conclusion

Using the QSAT, the findings from this single-site cohort suggest that faculty must be included in MSF. Self-evaluation appears to be of limited value in MSF with the QSAT. The degree of MSF agreement as gathered by the QSAT was lower in this cohort than previously reported for adult simulation cases performed in the simulation lab. This may be due to either the pediatric nature of the case, the location of the simulation, or both.

## Introduction

Pediatric (Peds) simulation using a simulation mannequin can help to assess a learner’s progress through residency training on a variety of emergency department (ED) case presentations while in a controlled, structured environment. Since its initiation, simulation has evolved in its application to include not only teaching but also to be a means for assessment. Assessment of some residency core competencies has been found to be better conducted through simulation than other traditional means [1]. The Accreditation Council for Graduate Medical Education (ACGME) requires the assessment of emergency medicine (EM) residents through a set of milestones, and simulation has been deemed an acceptable form of assessment specifically for Milestones 1-11 and 16-23 [2].

There are several different proposed tools to evaluate EM learners in the simulation environment [3-5], but few of them have been validated in multicenter studies like the Queen’s Simulation Assessment Tool (QSAT) [6]. First described in 2012, the QSAT was developed via a Delphi process with the intent of being "simple and modifiable" to new simulation cases [6,7]. The QSAT measures four performance domains (initial assessment, diagnostic approach, therapeutic approach, and communication skills) in addition to providing a single global assessment [7]. These five categories are all measured on a 5-point scale [7]. The QSAT was validated for resident assessment in resuscitation cases and was able to differentiate between post-graduate year (PGY) classes by demonstrating persistently higher scores among senior residents [8]. In these studies, the QSAT was completed exclusively by EM faculty [6-8].

According to the ACGME, multi-source feedback (MSF) is a suggested method of evaluation for 10 of the 23 Milestones [2]. Data for MSF is drawn from learners being evaluated by peers, colleagues, and/or supervisors. Several studies have demonstrated adequate reliability using simulation performance checklists to generate MSF [9-11].

New research continues to be conducted regarding the use of MSF combined with simulation. Previous research using the QSAT for MSF has shown favorable outcomes with excellent inter-rater reliability (IRR) in an adult simulation case in the simulation lab [12]. However, the QSAT has not yet been studied in a Peds simulation conducted in the clinical environment (in situ). In situ simulation has been shown to have benefits over simulation in the simulation lab, which include teamwork, patient safety, cost, availability, repetition, and realistic setting [13,14]. Our study aimed to determine the concordance of rater evaluations of the QSAT when used to provide MSF to assess EM resident performance in a single, standardized, in situ Peds simulation resuscitation case performed within a children’s ED.

## Materials and methods

This prospective study was an a priori planned extension of a two-part study. Part one of the Institutional Review Board (IRB) protocol entailed the simulation of an adult toxic ingestion case conducted in the simulation lab. These MSF results were previously published [12]. This study represents part two of the IRB protocol and evaluates the concordance of MSF for a Peds simulation case conducted in situ. Similar to the QSAT development studies, a single Peds resuscitation case was developed by a group of simulation-trained EM physicians using standard simulation templates [6-8]. The case, a toxic ingestion, was run using a high-fidelity Peds simulation mannequin in a patient room within the children’s ED. All simulations were conducted during a single standard in situ time: 0700 to 0730 on Tuesdays. A maximum of two simulations was conducted in any single calendar month.

The study was conducted at a four-year dually approved EM residency at an independent academic center within a suburban healthcare network. The program trains 14 residents per PGY. All study participants consented to participation in the simulation. As part of the consent process, an independent party’s contact information was provided. This party, an educator within the Network’s Department of Education, could be contacted by study subjects to allow for anonymous removal from the study.

All EM residents in their PGY 2-4 levels of training were eligible for enrollment as team leaders. The team leader was provided MSF using the QSAT, a previously validated rubric [6-8]. The QSAT assesses resident performance in four categories using case-specific behavioral anchors: primary assessment of the patient; diagnostic testing; treatment of the underlying condition; and interpersonal communication with staff and consultants. The QSAT with the case-specific behavioral anchors is shown in Table 1. A fifth category, overall assessment, is both holistic and was left to the interpretation of the assessor.

**Table 1:** Queen’s Simulation Assessment Tool (QSAT) With Behavioral Anchors

Primary Assessment					
Vital signs: heart rate (HR)/blood pressure (BP)/O_2_ saturation/respiratory rate (RR)/temperature + glucose cardiac monitor intravenous access			Level of consciousness (LOC) assessment, airway assessment, and rhythm assessment		
1	2	3		4	5
Delayed or incomplete performance of all criteria	Delayed or incomplete performance of many criteria	Delayed or incomplete performance of some criteria		Competent performance of most criteria	Competent performance of all criteria

Diagnostic Actions					
History: allergies, history of present illness (HPI), past medical history (PMHx), medications, physical exam, electrocardiogram (ECG)			Bloodwork: Acetaminophen (APAP) (Tylenol), arterial blood gas (ABG), ASA (aspirin) levels, complete blood count (CBC), comprehensive metabolic panel (CMP), point of care (POC) glucose, post-intubation chest X-ray (CXR)		
1	2	3		4	5
Delayed or incomplete performance of all criteria	Delayed or incomplete performance of many criteria	Delayed or incomplete performance of some criteria		Competent performance of most criteria	Competent performance of all criteria

Therapeutic Actions					
Supplemental O_2_ with bag valve mask (BVM), rapid sequence intubation with meds			Naloxone resuscitation with age-appropriate fluid bolus		
1	2	3		4	5
Delayed or incomplete performance of all criteria	Delayed or incomplete performance of many criteria	Delayed or incomplete performance of some criteria		Competent performance of most criteria	Competent performance of all criteria

Communication					
Introduces self and explains the clinical situation; clear and concise orders and direction; prioritizes tasks and anticipates further steps			Demonstrates leadership in managing crisis; appropriate specialist consultation: toxicologist, pediatric intensive care unit		
1	2	3		4	5
Delayed or incomplete performance of all criteria	Delayed or incomplete performance of many criteria	Delayed or incomplete performance of some criteria		Competent performance of most criteria	Competent performance of all criteria

Overall Assessment				
1	2	3	4	5
Delayed or incomplete performance of all criteria	Delayed or incomplete performance of many criteria	Delayed or incomplete performance of some criteria	Competent performance of most criteria	Competent performance of all criteria

MSF was provided to the team leader from five sources: two attendings, two nurses, and a resident peer. The team leader, through self-assessment, provided the sixth source. As with part one, in order to control for issues of multiple comparisons, each source of MSF contributed in proportions determined a priori [11]. For the attendings, there was a single fixed EM core teaching faculty member present at all simulations and an EM faculty dyad comprising two core faculty who participated in 50% of the cases each. From nursing [registered nurse (RN)], MSF was provided by a random, general EM nurse (ED RN) enrolled once and only once, and a fixed Peds EM-trained nurse (Peds RN) was present at all simulations. The peer resident, enrolled once and only once, was intentionally junior (as measured by PGY) to the team leader. Each team leader, enrolled once for the study, performed a self-evaluation.

The QSAT was completed by all participants in the patient room utilized for the simulation as soon as the case was completed. While no prior training on the QSAT was provided to the study participants, the evaluating faculty had experience in developing the simulation case and QSAT anchors. Participants provided personal demographics prior to the simulation. PGY was based on the date of enrollment.

Data analysis

The analytic plan has been previously described [12]. Descriptive statistics described the sample, and both counts and percentages were used for categorical variables. For normally distributed continuous data, results were presented via mean and standard deviation. Median was used for non-normal distributions. Normalcy was determined by skew of less than +1 and greater than -1 via visual inspection of the histogram plots. The plan to avoid issues with repeated measures is presented above.

The cumulative QSAT score was the sum of the rating in each of the five sections, resulting in totals ranging from 5-25. As in part one, hypothesis testing was assessed via IRR by obtaining intraclass correlation coefficients (ICC) for the groups of raters [12]. Two-way random ICCs were used to determine the average level of absolute rater agreement between all raters within each simulation. ICC interpretation is as per Cicchetti, who defined results less than 0.40 as poor, 0.40 to 0.59 as fair, 0.60 to 0.74 as good, and ≥0.75 as excellent [15]. ICC was calculated systematically across the different sources of MSF.

All analyses were two-tailed with the alpha set at 0.05. All statistical analyses were performed using SAS version 9.3 (SAS Institute, Cary, NC) and SPSS Statistics version 24 for Windows (IBM, Armonk, NY).

## Results

This study enrolled 106 participants prospectively during 35 separate in situ Peds simulation over the course of two academic years. Enrollees included three faculty members, 35 team leaders, 34 peers, 33 ED RN, and one Peds RN; 53 of the enrollees were female (50%). Team leaders were Doctor of Osteopathic Medicine (DO) (86%, n=30) and Doctor of Medicine (MD) (14%, n=5). The PGY of team leaders was as follows: three PGY 2, 18 PGY 3, and 14 PGY 4. Peers consisted of DO (85%, n=29) and MD (15%, n=5). The median years of experience for ED RN was 2.5 (IQR: 1.0-9.0).

Table 2 reports, for each source of MSF, the average QSAT for each QSAT Category, along with the total score. For the entire cohort, self-evaluations accounted for the lowest summative scores, and the lowest categorical scores for Primary assessment, Diagnostic Action (tied), and Overall Assessment. Self-evaluation was followed closely by the fixed faculty, who had the lowest categorical scores in Diagnostic Action (tied), Therapeutic Actions, and Communication. Peer evaluations accounted for the highest summative scores and were the highest in each of the five categorical scores. These differences, based on 95% CI, were not statistically significant.

Table 2 also includes the scores based on the PGY status of the team leader. Scores tended to be higher with higher PGY year status, but this trend was neither consistent nor statistically significant. Self-evaluation had the lowest total score for both PGY 3 and PGY 4. Peer evaluations had the highest summative score for all PGY levels.

**Table 2:** Average QSAT Scores by MSF Source for Entire Cohort and Stratified by PGY of Resident Team Leader ^a^One self-rater did not answer the overall assessment question; QSAT total unable to be calculated for simulation, n=34 ^b^One simulation is missing data from a peer rater, n=34 ^c^Two simulations are missing data from the static Peds RN rater, n=33 ^d^One simulation is missing data from the dynamic ED RN raters, n=34 ^e^One self-rater did not answer the overall assessment question; QSAT total unable to be calculated for the simulation, n=17 ^f^One simulation missing data from peer rater, n=17 ^g^Two simulations missing from static Peds RN rater, n=12 ^h^One simulation missing from dynamic ED RN rater, n=17 MSF: multi-source feedback; PGY: post-graduate year; Peds: pediatric; QSAT: Queen’s Simulation Assessment Tool; RN: registered nurse; SD: standard deviation

Average QSAT Scores by Rater for the Entire Cohort (N=35 Unless Noted)						
QSAT Variable (Mean ± SD)	Self-Evaluation	Fixed Faculty	Faculty Dyad	Peer Resident^b^	Peds RN^c^	ED RN^d^
Primary Assessment	4.2 ± 0.6	4.4 ± 0.7	4.4 ± 0.7	4.8 ± 0.4	4.5 ± 0.8	4.7 ± 0.5
Diagnostic Actions	4.0 ± 0.7	4.0 ± 0.8	4.3 ± 0.7	4.4 ± 0.6	4.2 ± 0.9	4.3 ± 0.7
Therapeutic Actions	4.3 ± 0.7	4.2 ± 0.8	4.5 ± 0.8	4.8 ± 0.5	4.2 ± 0.9	4.5 ± 0.6
Communication	4.3 ± 0.7	4.2 ± 0.8	4.6 ± 0.6	4.7 ± 0.5	4.4 ± 0.7	4.4 ± 0.7
Overall Assessment	4.0 ± 0.6^a^	4.4 ± 0.6	4.2 ± 0.5	4.7 ± 0.5	4.4 ± 0.7	4.4 ± 0.6
QSAT Total	20.7 ± 2.6^a^	21.2 ± 2.5	22.3 ± 1.9	23.4 ± 1.9	21.7 ± 3.1	22.4 ± 2.4
Average QSAT Scores by Rater for PGY 4 Resident Team Leader (N=14 Unless Noted)						
Primary Assessment	4.2 ± 0.6	4.4 ± 0.7	4.4 ± 0.7	4.8 ± 0.4	4.6 ± 0.8^g^	4.9 ± 0.4
Diagnostic Actions	3.9 ± 0.7	4.0 ± 0.8	4.4 ± 0.7	4.5 ± 0.7	4.3 ± 1.0^g^	4.3 ± 0.6
Therapeutic Actions	4.2 ± 0.7	3.9 ± 0.7	4.4 ± 0.8	4.7 ± 0.6	3.8 ± 0.9^g^	4.6 ± 0.5
Communication	4.1 ± 0.6	4.2 ± 0.7	4.7 ± 0.5	4.7 ± 0.5	4.3 ± 0.9^g^	4.4 ± 0.6
Overall Assessment	4.1 ± 0.5	4.4 ± 0.5	4.3 ± 0.5	4.8 ± 0.4	4.3 ± 0.8^g^	4.4 ± 0.5
QSAT Total	20.5 ± 2.7	20.9 ± 2.3	22.5 ± 1.5	23.5 ± 2.1	21.3 ± 3.9^g^	22.4 ± 1.9
Average QSAT Scores by Rater for PGY 3 Resident Team Leader (N=18 Unless Noted)						
Primary Assessment	4.1 ± 0.6	4.4 ± 0.6	4.4 ± 0.6	4.8 ± 0.4^f^	4.5 ± 0.8	4.6 ± 0.6^h^
Diagnostic Actions	4.1 ± 0.6	4.0 ± 0.7	4.2 ± 0.7	4.3 ± 0.5^f^	4.1 ± 0.8	4.4 ± 0.7^h^
Therapeutic Actions	4.3 ± 0.8	4.4 ± 0.8	4.6 ± 0.7	4.9 ± 0.3^f^	4.4 ± 0.7	4.5 ± 0.7^h^
Communication	4.3 ± 0.8	4.3 ± 0.9	4.6 ± 0.6	4.8 ± 0.4^f^	4.5 ± 0.6	4.5 ± 0.9^h^
Overall Assessment	3.9 ± 0.6^e^	4.4 ± 0.6	4.1 ± 0.6	4.7 ± 0.5^f^	4.4 ± 0.6	4.5 ± 0.6^h^
QSAT Total	20.7 ± 2.7^e^	21.6 ± 2.6	22.2 ± 2.1	23.4 ± 1.8^f^	22.0 ± 2.6	22.5 ± 2.9^h^
Average QSAT Scores by Rater for PGY 2 Resident Team Leader (N=3 Unless Noted)						
Primary Assessment	4.7 ± 0.6	4.7 ± 0.6	4.7 ± 0.6	5.0 ± 0.0	4.0 ± 1.0	5.0 ± 0.0
Diagnostic Actions	3.7 ± 0.6	3.7 ± 1.2	4.3 ± 0.6	4.0 ± 1.0	4.7 ± 0.6	3.7 ± 1.2
Therapeutic Actions	4.3 ± 0.6	4.3 ± 1.2	4.3 ± 1.2	4.7 ± 0.6	4.0 ± 1.0	4.3 ± 0.6
Communication	4.7 ± 0.6	3.7 ± 0.6	4.0 ± 1.0	4.7 ± 0.6	4.3 ± 0.6	4.0 ± 0.0
Overall Assessment	4.0 ± 1.0	4.0 ± 1.0	4.3 ± 0.6	4.3 ± 0.6	4.7 ± 0.6	4.3 ± 0.6
QSAT Total	21.3 ± 2.1	20.3 ± 4.0	21.7 ± 2.9	22.7 ± 2.1	21.7 ± 3.2	21.3 ± 2.3

Of the 35 simulations, 30 had complete evaluations by all six sources of MSF. The average total scores by MSF for those 30 simulations are presented in Table 3. All ratings are negatively skewed, indicating that most scores were high with a few, outlying low scores.

**Table 3:** QSAT Score Descriptive Statistics for Sim with Complete MSF (N=30) ED: emergency department; MSF: multi-source feedback; Peds: pediatric; QSAT: Queen’s Simulation Assessment Tool; RN: registered nurse; SD: standard deviation

Rater	Mean	Standard Deviation	Median	Interquartile Range	Mode	Range	Skew
Self	20.6	2.7	20.5	(19.0-22.3)	20.0	(15.0-25.0)	-0.34
Fixed Faculty	21.3	2.3	21.0	(19.0-24.0)	24.0	(16.0-24.0)	-0.32
Faculty Dyad	22.5	1.9	23.0	(21.0-24.0)	23.0	(18.0-25.0)	-0.46
Peer Resident	23.5	1.7	24.0	(21.8-25.0)	25.0	(20.0-25.0)	-0.83
Peds RN	21.6	3.2	23.0	(19.0-24.0)	24.0	(14.0-25.0)	-0.85
ED RN	22.3	2.5	23.0	(20.8-24.0)	24.0	(15.0-25.0)	-0.99

The ICC in Table 4 demonstrates the degree of agreement between sources of MSF. This agreement analysis speaks to whether each source of MSF provided the same score as the other sources. Based on definitions by Cichetti, this agreement for all raters was fair at 0.531 (ICC 1) [15]. Table 4 also demonstrates the impact of systematically removing each source of MSF. Removing faculty evaluations led to the lowest inter-rater agreement (ICC 6 = 0.364). The removal of self-evaluation (ICC 5 = 0.579) led to the highest levels of agreement among the remaining sources of MSF.

**Table 4:** Intraclass Correlation Coefficients (ICC) for Inter-Rater Reliability of Mean Total QSAT Score ^1^ICC for all raters ^2^ICC with Peds RN rater removed ^3^ICC with peer raters removed ^4^ICC coefficient with ED RN raters removed ^5^ICC with self raters removed ^6^ICC with all faculty raters removed ED: emergency department; Peds: pediatric; QSAT: Queen’s Simulation Assessment Tool; RN: registered nurse; CI: confidence interval

ICC Type	All^1^	Faculty, Self, Peer, ED RN^2^	Faculty, Self, ED RN, Peds RN^3^	Faculty, Self, Peer, Peds RN^4^	Faculty, Peer, ED RN, Peds RN^5^	Self, Peer, ED RN, Peds RN^6^
Absolute Agreement, Average Measures (95% CI)	0.531 (0.244-0.742)	0.377 (0.017-0.651)	0.538 (0.232-0.751)	0.488 (0.173-0.718)	0.579 (0.303-0.772)	0.364 (-0.027-0.650)

Table 5 demonstrates that the ICC for faculty alone (without other sources of MSF) was fair at 0.540. Removing faculty as a source of MSF led to the lowest ICC (0.364, 95% CI: -0.027-0.650). Of note, the confidence interval crossed zero. While the faculty agreement was fair, combining the faculty with other sources of MSF did not improve the degree of agreement.

**Table 5:** Agreement of Total QSAT Scores Between Faculty and Other Sources of MSF ED: emergency department; ICC: intraclass correlation coefficient; MSF: multi-source feedback; Peds: pediatric; QSAT: Queen’s Simulation Assessment Tool; RN: registered nurse

ICC Type	Faculty	All Other Raters	Faculty + Peer	Faculty + Peds RN	Faculty + ED RN
Absolute Agreement, Average Measures (95% CI)	0.540 (0.071-0.777)	0.364 (-0.027-0.650)	0.349 (-0.070-0.644)	0.532 (0.162-0.759)	0.494 (0.102-0.738)

## Discussion

The agreement between sources of MSF using the QSAT in this cohort was both fair at best and lower than we had previously reported [12]. The lesser agreement may be due to the simulation being a Peds case, an in situ simulation, or both.

Exposure to Peds resuscitation cases has been noted to be low for trainees [16]. The limited number of reliable and validated Peds simulation assessment tools has been previously noted [17]. A study of emergency medical services (EMS) providers found a longer time to intervention in Peds vs. adult simulation case, though the difference was not statistically significant [18]. A study of adult EM physicians observed that pediatric skill begins to decrease after six months [19]. So while the increased frequency of Peds critical care education offered may be a counter-measure to lower skills, it may not improve inter-rater agreement for MSF for Peds cases. In fact, in this cohort, the negative skew in Table 3 demonstrates that MSF raters more frequently scored participants highly. This suggests that the decreased inter-rater agreement is likely more closely linked to confounders other than Peds critical care skills.

Another explanation is the in situ nature of this cohort. While in situ simulation may have its benefits, there are unique challenges as well [13,14]. In situ simulation has been described as having different goals from the simulations offered in the simulation lab [20]. Here, "time pressures" are noted as a barrier to in situ simulations. Difficulties in locating eligible participants from available clinical staff led to incomplete sources of MSF as noted in Table 2. Since the enrollees, except for the faculty, needed to return to clinical duties, this may have impacted their QSAT scores. The impact of "time pressure" is complex and has been the source of study in the sociology literature [21]. Issues noted include a "speed-accuracy trade-off" and that people will cope by "attend(ing) selectively to information." That said, if MSF is to be utilized in the clinical environment, staff and faculty alike will experience some degree of "time pressure." If the impact of "time pressure" is one of decreased agreement, the ability of Clinical Competency Committees (CCC) to utilize MSF to evaluate residents based on clinical encounters may be problematic. The ACGME recommends MSF for Milestone 6 (Observation and Re-Assessment), 7 (Disposition), 8 (Multi-Tasking), all of which are Patient Care Competencies [2]. MSF may be possible for Systems-Based Practice (Milestones 16, 17, 18), Professionalism Competencies (Milestone 20, 21), and Communication Competencies (Milestone 22 and 23) [2] where MSF may be obtained in real time outside of the temporal pressures of the ED. Otherwise, if the decreased agreement was in fact due to "time pressure," MSF would need to be obtained summatively rather than in real time.

This study is limited by the fact that the Peds case was internally developed. The negative skew noted in Table 3 may have occurred because the case was "too easy." This conclusion is further supported by the fact that Table 2 does not demonstrate a clear progression of scores as PGY increases. Since the study’s hypothesis was to test the degree of agreement between different sources of MSF, not to create a simulation case that differentiates the abilities of the enrolled resident team leaders, the difficulty of the case should not impact the degree of agreement. The negative skew may have artificially increased the agreement seen since the lower part of the 1-5 scale was used with decreased frequency. Even though in Table 2 there is not a significant increase in total QSAT scores as PGY increases, it is interesting to note that as PGY increases, self-evaluation total scores decrease. This finding appears consistent with the "Dunning-Kruger effect" in which the less skilled overestimate their abilities in large part because of a lack of understanding or "irrational optimism." [22] Concordant with our initial findings, self-assessment decreased the correlation of MSF in this cohort as well [12]. Table 4 demonstrates that the greatest agreement occurs when the self rater is removed. Milestone 19, Level 3 anchor notes that programs must ask the resident to self-assess [2]. The self-assessment behavioral anchor lies within the Problem-Based Learning competency, which also houses Evidence-Based Medicine (EBM) skills. It has been demonstrated that self-assessment of information literacy (EBM) skills has "little calibration" with actual skill [23]. In the end, the process of self-assessment may be better done holistically or summatively rather than on an individual case. At the very least, this data reinforces the fact that the QSAT is not an appropriate instrument for self-assessment.

In this cohort, the total and categorical scores by peer evaluator were the highest overall and for each stratified PGY except for PGY 4 primary assessment, and the PGY 2 and 3 diagnostics. Peer raters had the highest, or tied for the highest, score in 21 of the 24 possible categorical and total scores. A previous study has found that providing "uphill" feedback is perceived as difficult for junior residents [24]. Concerns about the personal relationships of the residents were also noted. These high scores on the QSAT are in contrast to our prior work where peers were almost universally lower than not just both faculty scores but were occasionally lower than the EMS score [12]. Because this part of the larger study protocol followed the first, it may have impacted the peer residents. The location (in situ while on shift vs. simulation lab during grand rounds) and duration of the study (the initial study was completed in weeks; this study spanned over two academic years) may have also played a role. While the two cohorts demonstrate a different relationship between peer and faculty (ICC of 0.799 previously [12], here in Table 5, 0.349), there is evidence that peer evaluations have a positive impact on performance [25]. For residents, peer evaluation of teaching in the clinical environment has been demonstrated to be feasible, suggesting that in certain circumstances, peer contributions to MSF on shift may be possible [26]. Peer contribution to MSF for the Professionalism and Communication competencies was determined to be both possible and reliable [27]. As noted above, for EM, these ACGME competencies may prove to be the most practical to focus on in future studies of MSF.

In the end, the decreased level of agreement in this study may most closely correlate with the decreased agreement between the faculty. Here the agreement between the faculty was 0.570 (Table 5) as compared to 0.840 previously [12]. While the fixed faculty had lower scores than the dyad (Table 2), the 95% CI always overlapped. Interestingly, the 95% CI drops into negative values when the faculty raters are removed (Table 5). Negative ICCs are theoretically difficult to interpret. Some argue that they are invalid (however still mathematically computational, which is why they are calculated), while others argue that they are valid and it just signifies disagreement/poor agreement [28,29]. By all measures, this cohort again confirms that faculty is a necessary component of MSF, and their inclusion yields the highest level of agreement. The 95% CI also drop into negative numbers when faculty and peer are combined. This stems from the elevated peer scores discussed above combined with the lower scores provided by the fixed attending. While conducted at the same program, the enrolled faculty providing scores did not overlap in the two studies. Taken together, the two cohorts appear to support the important role of the CCC. The ACGME notes that "CCC can be an opportunity to balance out the 'hawks' and 'doves.'" [30] For programs attempting to utilize the QSAT for MSF either in the simulation lab, for in situ simulation, or for actual patient encounters, the CCC should interpret the findings based on their experience with the faculty who contribute the scores.

Limitations

Limitations of this study include its small sample size and single site of enrollment. While the QSAT has been previously validated, the Peds simulation case used here was not [6-8]. Not every Peds simulation conducted had all six sources of MSF. Consistent with prior work on using the QSAT for MSF, the team members did not receive explicit QSAT training [12]. Training, described as simple, was provided in prior faculty study of the QSAT [6-8]. This lack of training, while felt to better reflect how the QSAT would be employed for in situ MSF, or for MSF on actual patient encounters, may have impacted the results. Finally, the impact of the pediatric nature of the case and the in situ nature of the simulation cannot be separated. The case, the location, or both, may have impacted the results.

## Conclusions

In this single-site simulation study using a locally developed Peds toxic ingestion case, the QSAT demonstrated fair inter-rater agreement. This level of agreement may have been influenced by the in situ nature of the study, the pediatric nature of the case, or both. Unless faculty are included in QSAT MSF, the agreement is poor, with a mathematical suggestion of disagreement. Peer evaluation consistently provided the highest scores in this cohort, though the differences between the sources of MSF were not significant. When using the QSAT, self-evaluation decreases agreement, suggesting that this form of MSF may not be appropriate with this instrument. Reliable ways to gather MSF to inform CCCs remains a challenge for EM, and future efforts may be best focused on ACGME Competencies other than Patient Care.

## References

[1] Mallory LA, Calaman S, Lee White M (2016). Targeting simulation-based assessment for the pediatric milestones: a survey of simulation experts and program directors. Acad Pediatr.

[2] (2020). The Emergency Medicine Milestone Project. https://www.acgme.org/Portals/0/PDFs/Milestones/EmergencyMedicineMilestones.pdf.

[3] Levy A, Donoghue A, Bailey B, Thompson N, Jamoulle O, Gagnon R, Gravel J (2014). External validation of scoring instruments for evaluating pediatric resuscitation. Simul Healthc.

[4] Nadkarni LD, Roskind CG, Auerbach MA, Calhoun AW, Adler MD, Kessler DO (2018). The development and validation of a concise instrument for formative assessment of team leader performance during simulated pediatric resuscitations. Simul Healthc.

[5] Wieck MM, McLaughlin C, Chang TP (2018). Self-assessment of team performance using T-NOTECHS in simulated pediatric trauma resuscitation is not consistent with expert assessment. Am J Surg.

[6] Dagnone JD, Hall AK, Sebok-Syer S (2016). Competency-based simulation assessment of resuscitation skills in emergency medicine postgraduate trainees - a Canadian multi-centred study. Can Med Educ J.

[7] Hall AK, Pickett W, Dagnone JD (2012). Development and evaluation of a simulation-based resuscitation scenario assessment tool for emergency medicine residents. CJEM.

[8] Hall AK, Dagnone JD, Lacroix L, Pickett W, Klinger DA (2015). Queen's simulation assessment tool: development and validation of an assessment tool for resuscitation objective structured clinical examination stations in emergency medicine. Simul Healthc.

[9] Wright C, Richards SH, Hill JJ (2012). Multisource feedback in evaluating the performance of doctors: the example of the UK General Medical Council patient and colleague questionnaires. Acad Med.

[10] Donnon T, Al Ansari A, Al Alawi S, Violato C (2014). The reliability, validity, and feasibility of multisource feedback physician assessment: a systematic review. Acad Med.

[11] Garra G, Wackett A, Thode H (2011). Feasibility and reliability of a multisource feedback tool for emergency medicine residents. J Grad Med Educ.

[12] Jong M, Elliott N, Nguyen M (2019). Assessment of emergency medicine resident performance in an adult simulation using a multisource feedback approach. West J Emerg Med.

[13] Petrosoniak A, Auerbach M, Wong AH, Hicks CM (2017). In situ simulation in emergency medicine: moving beyond the simulation lab. Emerg Med Australas.

[14] Truchot J, Boucher V, Raymond-Dufresne É (2021). Evaluation of the feasibility and impacts of in situ simulation in emergency medicine-a mixed-method study protocol. BMJ Open.

[15] Cicchetti DV (1994). Guidelines, criteria, and rules of thumb for evaluating normed and standardized assessment instruments in psychology. Psychol Assess.

[16] Lin Y, Cheng A (2015). The role of simulation in teaching pediatric resuscitation: current perspectives. Adv Med Educ Pract.

[17] Weinberg ER, Auerbach MA, Shah NB (2009). The use of simulation for pediatric training and assessment. Curr Opin Pediatr.

[18] Khalil PA, Berkovich J, Maniaci V, Lozano JM, Lowe DA (2020). Performance of emergency medical service providers in pediatric and adult simulation of unstable supraventricular tachycardia. Pediatr Emerg Care.

[19] Ansquer R, Mesnier T, Farampour F, Oriot D, Ghazali DA (2019). Long-term retention assessment after simulation-based-training of pediatric procedural skills among adult emergency physicians: a multicenter observational study. BMC Med Educ.

[20] Patterson MD, Blike GT, Nadkarni VM (2020). In situ simulation: challenges and results. Advances in Patient Safety: New Directions and Alternative Approaches.

[21] Ordóñez LD, Benson III L, Pittarello A (2015). Time pressure perception and decision making. The Wiley Blackwell Handbook of Judgment and Decision Making, II.

[22] Simons DJ (2013). Unskilled and optimistic: overconfident predictions despite calibrated knowledge of relative skill. Psychon Bull Rev.

[23] Mahmood K (2016). Do people overestimate their information literacy skills? A systematic review of empirical evidence on the Dunning-Kruger effect. Commun Inform Literacy.

[24] de la Cruz MS, Kopec MT, Wimsatt LA (2015). Resident perceptions of giving and receiving peer-to-peer feedback. J Grad Med Educ.

[25] Double KS, McGrane JA, Hopfenbeck TN (2020). The impact of peer assessment on academic performance: a meta-analysis of control group studies. Educ Psychol Rev.

[26] Snydman L, Chandler D, Rencic J, Sung YC (2013). Peer observation and feedback of resident teaching. Clin Teach.

[27] Zhao Y, Zhang X, Chang Q, Sun B (2013). Psychometric characteristics of the 360° feedback scales in professionalism and interpersonal and communication skills assessment of surgery residents in China. J Surg Educ.

[28] Hallgren KA (2012). Computing inter-rater reliability for observational data: an overview and tutorial. Tutor Quant Methods Psychol.

[29] Giraudeau B (1996). Negative values of the intraclass correlation coefficient are not theoretically possible. J Clin Epidemiol.

[30] 27]. Accreditation Council for Graduate Medical Education (2020). Accreditation Council for Graduate Medical Education. https://www.acgme.org/Portals/0/ACGMEClinicalCompetencyCommitteeGuidebook.pdf.

